# Is there a threshold level of maternal education sufficient to reduce child undernutrition? Evidence from Malawi, Tanzania and Zimbabwe

**DOI:** 10.1186/s12887-015-0406-8

**Published:** 2015-08-22

**Authors:** Donald Makoka, Peninah Kinya Masibo

**Affiliations:** Centre for Agricultural Research and Development (CARD), Lilongwe University of Agriculture and Natural Resources, P.O. Box 219, Lilongwe, Malawi; School of Public Health, Department of Nutrition, Moi University, Nairobi campus, P.O. Box 63056 - 00200, Nairobi, Kenya; African Population and Health Research Center (APHRC), Research Capacity Strengthening, Training Programs, Kirawa Road, Off Peponi Road, P.O. Box 10787-00100, Nairobi, Kenya

**Keywords:** Maternal education, Threshold level, Child undernutrition, Demographic health survey, Malawi, Tanzania, Zimbabwe

## Abstract

**Background:**

Maternal education is strongly associated with young child nutrition outcomes. However, the threshold of the level of maternal education that reduces the level of undernutrition in children is not well established. This paper investigates the level of threshold of maternal education that influences child nutrition outcomes using Demographic and Health Survey data from Malawi (2010), Tanzania (2009–10) and Zimbabwe (2005–06).

**Methods:**

The total number of children (weighted sample) was 4,563 in Malawi; 4,821 children in Tanzania; and 3,473 children in Zimbabwe Demographic and Health Surveys. Using three measures of child nutritional status: stunting, wasting and underweight, we employ a survey logistic regression to analyse the influence of various levels of maternal education on child nutrition outcomes.

**Results:**

In Malawi, 45 % of the children were stunted, 42 % in Tanzania and 33 % in Zimbabwe. There were 12 % children underweight in Malawi and Zimbabwe and 16 % in Tanzania.The level of wasting was 6 % of children in Malawi, 5 % in Tanzania and 4 % in Zimbabwe. Stunting was significantly (p values < 0.0001) associated with mother’s educational level in all the three countries. Higher levels of maternal education reduced the odds of child stunting, underweight and wasting in the three countries. The maternal threshold for stunting is more than ten years of schooling. Wasting and underweight have lower threshold levels.

**Conclusion:**

These results imply that the free primary education in the three African countries may not be sufficient and policies to keep girls in school beyond primary school hold more promise of addressing child undernutrition.

## Background

Child undernutrition is a persistent health challenge worldwide and especially in the developing countries where one child in three is stunted. Undernutrition accounts for 35% of annual deaths for children less than 5 years of age[1, 2]. Children who survive are more vulnerable to infection, do not reach their full height potential and experience impaired cognitive development among other complications. Without intervention undernutrition can continue throughout the life cycle [3].

The importance of a mother’s education on child health and nutrition has been well demonstrated in a number of studies [4–8]. Mother’s education is associated with better children’s health and nutritional outcomes through improving the socioeconomic status of mothers [9]. In turn, the higher socioeconomic status mitigates a set of proximate determinants of health that directly influences on the health and nutritional outcomes of children. The proximate determinants include fertility factors, feeding practices and the utilization of health services [10]. It is argued that maternal education improves the mother’s knowledge about child health, including causes, prevention and treatment of diseases [11].

This paper assesses the relationship between maternal education and child undernutrition in Malawi, Tanzania and Zimbabwe. The three countries share a number of common characteristics in terms of geographical location and socioeconomic factors. However, while Malawi and Zimbabwe have educational systems that were modeled under the British educational system, Tanzania’s educational system uses Kiswahili language as the medium of instruction throughout primary school. The three countries therefore provide useful case studies on the relationship between maternal education and child nutritional outcomes.

Since the 1980s, there has been a drive to promote free primary education in Africa. For example, free primary education was introduced in Zimbabwe in 1980, Malawi in 1994 and in Tanzania in 2002 to promote literacy. One of the implicit assumptions in the promotion of free primary education is that improved literacy would lead to improved health-seeking behaviour and improved nutrition for the population. In particular, it has been argued that literate women are more likely to be aware of the importance of immunizing children against diseases, feeding the child at the appropriate time and in right quantities and taking early actions against diarrhea and other infant diseases [12]. Studies in various settings have shown association between child nutrition outcomes with maternal education [9, 11, 13]. Literature on modeling the associations of maternal education on child nutrition outcomes has mainly focused on four non-excluded models: socio-economic status; women empowerment and autonomy; health knowledge and attitudes; and health and reproductive behaviour [14]. Studies that use maternal education as a proxy for socio-economic status both at the individual and household levels, argue that more educated women tend to have better work opportunities and they are more likely to marry more educated husbands [15]. More educated women also tend to live in urban areas where they have access to better health and sanitation services. Despite the available of evidence on the influence of maternal education on child nutrition outcomes, it is not clear the threshold of maternal education that is required for cause a positive outcome on child nutritional status. This raises the question on whether introduction of free primary education to increase literacy levels in sub-Saharan Africa has any influence on improved child nutrition.

It is against this background that we analyzed nationally representative data from three sub-Saharan African countries to explore the minimum level of maternal education that is sufficient to promote child nutrition.

## Methods

We analyzed data from the Demographic and Health Surveys (DHS) data collected in three southern African countries: Malawi (2010 dataset), Tanzania (Tanzania, 2009-10 dataset) and Zimbabwe (2005–06 dataset). The most recent dataset for Zimbabwe was not available at the time that this analysis was done. The three countries were selected conveniently based on the author’s prior knowledge on the Countries. Permission to use the DHS data from the three countries was granted by ICF International through the DHS Junior Faculty Fellowship Programme that both authors participated in.

The DHS are nationally representative sample of women aged 15 to 49 and their family members. Among others, the DHS surveys collect data on anthropometric indicators to provide outcome measures of nutritional status of under-five children and women. The study only considered data on children aged 0–59 months belonging to interviewed, *de facto* women whose weight and heights were taken and their mothers were interviewed. There was a weighted sample of 4,563 children in Malawi; 4,821 children in Tanzania; and 3,473 children in Zimbabwe. The response rates for women age 15 – 49 years was 96.9 % in Malawi, 96 % in Tanzania and 90.2 % in Zimbabwe.

The dependent variable in this study was child nutritional status measured as stunting, wasting and underweight defined as height/length-for-age, weight-for-height and weight – for-age z-scores below −2 standard deviations (−2 SD) from the median of the World Health Organization (WHO) reference population. Anthropometric measurements in the DHS are taken with children wearing light clothing, without shoes with bathroom-type scales for weight and a length board/mat for height or length for children age <24 months.

The background variables considered for this study were residence (rural or urban), sex of the household head, household wealth index, source of drinking water and access to sanitation facilities. The wealth index is a socio-economic index used as an indicator of household wealth based on ownership of assets and consumer goods such as source of drinking water, type of toilet facility, type of fuel used for cooking, ownership of various goods among other household characteristics. A factor score generated through principal components analysis is allocated to each asset and the resulting asset score are standardized in a relation to a normal distribution. Households have been grouped into five categories based on the wealth index as poorest, poorer, middle, richer and richest.

Independent variables included in the analysis were child demographics (age, sex), child birth order, whether the child had diarrhea in the two weeks prior to the survey and whether the child was a multiple birth. Maternal characteristics included current age, education, and the number of children under the age of five the mother had. The choice of the background variables as well as the explanatory variables was influenced by the conceptual framework that links maternal education and child nutrition proposed by UNICEF (1998) [16].The framework stipulates that the possible pathway through which maternal education can influence nutritional outcomes of children is through skills acquisition that leads to improved knowledge about health care and nutritional knowledge.

Maternal education was a key factor of consideration and was categorized based on the total number of years of schooling for the mother. Five categories were used in the analysis, namely: No schooling, junior primary (1–4 years of schooling), senior primary (5–7 years for Tanzania and Zimbabwe), (5–8 years for Malawi), junior secondary (8–10 years for Tanzania and Zimbabwe), (9–10 years for Malawi), senior secondary and above (>10 years).

### Statistical analysis

Data was analyzed using Stata software version 13:0 at the descriptive, bivariate and multivariate levels. Person χ2 test was applied to test for association between child nutritional outcomes namely stunted, wasted or underweight and the independent variables.

Binary logistic regression models were used to assess the relationship between the three measures of child nutritional status and maternal education as well as other background and child variables. Using the survey logistic regression analysis, a threshold level of maternal education was determined as the level of maternal education below which the results are not statistically significant. Data from each of the three countries was analyzed separately.

## Results

In Malawi, 45 % of the children were stunted, while in Tanzania 42 % of the children were stunted, and in Zimbabwe 33 % were stunted. The level of wasting was 6 % in Malawi, 5 % in Tanzania, and 4 % in Zimbabwe (Table 1). The proportion of underweight children was highest in Tanzania (16 %), 12 % in Malawi and 12 % in Zimbabwe.

**Table 1:** Distribution of child nutritional status, demographic characteristics, household characteristics and maternal characteristics in Malawi (2010), Tanzania (2009–10) and Zimbabwe (2005–6)

	Malawi		Tanzania		Zimbabwe	
	Mean (*n*)	SD	Mean (*n*)	SD	Mean (*n*)	SD
Child Nutritional Status						
Stunted	0.44 (2008)	0.01	0.42 (2024)	0.01	0.33 (1146)	0.01
Wasted	0.04 (183)	0.00	0.05 (241)	0.00	0.06 (208)	0.00
Underweight	0.12 (548)	0.01	0.16 (771)	0.01	0.12 (417)	0.01
Child Demographic Factors						
Age of child (in months)	28.89	0.27	28.56	0.19	28.9	0.36
Whether the child is male	0.49	0.01	0.50	0.01	0.51	0.01
Size of the child at birth- Small	2.69	0.07	3.03	0.03	2.73	0.02
Child is of multiple birth	0.03	0.00	0.02	0.00	0.02	0.00
Birth order	3.60	0.05	3.89	0.05	2.88	0.05
Child Health Factors						
Had diarrhoea recently	0.15	0.01	0.15	0.01	0.13	0.01
Socioeconomic Factors						
Whether place of residence is rural	0.85	0.01	0.82	0.01	0.74	0.02
Female Household head	0.08	0.01	0.14	0.01	0.33	0.01
Total number of people in the household	5.94	0.05	7.23	0.19	6.05	0.09
Household with safe water	0.78	0.01	0.53	0.02	0.70	0.03
Household with improved toilet facility	0.17	0.01	0.14	0.01	0.54	0.02
Maternal Demographic Factors						
Mother’s current age	28.40	0.13	29.54	0.15	28.07	0.12
Number of under-five children	1.84	0.02	2.23	0.05	1.80	0.03
Currently lactating (1 = Yes)	0.65	0.01	0.62	0.01	0.50	0.01
Maternal education level						
Mother’s education –None	0.18	0.01	0.26	0.01	0.04	0.01
Mother’s education –Junior Primary	0.29	0.01	0.10	0.01	0.06	0.01
Mother’s education –Senior Primary	0.38	0.01	0.57	0.01	0.37	0.02
Mother’s education –Junior Secondary	0.08	0.01	0.04	0.00	0.50	0.03
Mother’s education –Senior Secondary +	0.07	0.01	0.03	0.00	0.03	0.00
Husband’s education (in single years)	8.05	0.29	6.25	0.17	9.47	0.26
N	4,563		4,821		3,473	

The average age of the sampled children in all the three countries was 29 months. The average age of mothers was 28 years, living in households with average household size of 6. The proportion of female household heads was highest for Zimbabwe (33 %) and lowest in Malawi (8 %). In all the three countries, the sample was predominantly rural, with 85 % of children in Malawi, and 74 % of the children in Zimbabwe living in the rural areas. Access to safe water was highest in Malawi (78 %) and lowest in Tanzania (53 %) while the proportion of households with improved toilet facilities was highest in Zimbabwe (54 %) and lowest in Tanzania (14 %) (Table 1).

The distribution of maternal education was varied in the three countries. There was a higher percentage of women with no education in Tanzania (26 %), followed by Malawi (18 %), and 4 % in Zimbabwe. In Zimbabwe, 53 % of the women had junior secondary school education and above. The proportion of women with similar educational attainment in Malawi was 15 % and in Tanzania 7 % (Table 1).

### Bivariate results

Stunting was significantly (p values < 0.0001) associated with mother’s educational level in all the three countries. Half of the children whose mothers had no education were stunted in Malawi, 45 % in Tanzania and 34 % in Zimbabwe. Child stunting was lowest among children whose mothers had senior secondary education and above in the three countries (Table 2).

**Table 2:** Percent of children < 5 years with stunting, wasting and underweight by Mother’s Educational Level, residence and child age in Malawi (2010), Tanzania (2009–10) and Zimbabwe (2005–6)

	Malawi (2010)			Tanzania (2009–10)			Zimbabwe (2000–06)		
	Height-for-Age	Weight-for-height	Weight-for-age	Height- for-Age	Weight-for-height	Weight-for-age	Height- for-Age	Weight-for-height	Weight-for-age
	(% below -2SD)	(% below -2SD)	(% below -2SD)	(% below -2SD)	(% below -2SD)	(% below -2SD)	(% below -2SD)	(% below -2SD)	(% below -2SD)
Mother’s education level									
No education	50.3	4.8	15.1	44.9	6.1	18.9	33.8	7	17.6
Junior Primary	46.9	0.4	12.9	46.3	6.4	17	34.5	7.3	11.6
Senior Primary	42.9	3.7	11.8	41.2	4	14.7	34.1	8.2	14.2
Junior Secondary	41.7	2.4	7.3	30.4	4.9	9.6	32.5	5.1	10.9
Senior Secondary+	29.9	2.1	6.7	12.5	3.8	5.8	16.7	1.5	1.3
Pearson χ^2^	46.9	7.6	26.1	97.1	16	40	14	18.1	25.4
*P value*	*0.000*	*0.23*	*0.01*	*0.000*	*0.02*	*0.000*	*0.07*	*0.01*	*0.000*
Place of Residence									
Urban	38.2	2.4	10.1	30.6	5	11.5	26.6	5.2	8.4
Rural	45.6	4.2	12.4	44	4.8	16.6	35.1	6.8	13.5
Pearson χ^2^	13.8	5.4	3	79.6	0.12	20.8	25.2	3.1	18.7
*P-Value*	*0.01*	*0.09*	*0.25*	*0.000*	*0.79*	*0.000*	*0.000*	*0.18*	*0.000*
Child age									
Less than 6 months	14.2	5.7	5.3	18.1	6.1	8.2	13.8	9.3	7.7
6-11 months	25.1	6.2	9.6	24.3	9.9	14.9	23.5	9	11.4
12-23 months	52.5	6.3	15.1	49.3	7	18.4	38.8	6.4	12.7
24-35 months	54.1	2.3	12.8	53.2	3.3	15.6	39.8	6.7	15.5
36-47 months	48.6	2.4	12.2	47.7	2	15.8	39.5	4.8	11.8
48-59 months	46.1	2	12	37.7	3	16.9	28.5	4.5	11.6
Pearson χ^2^	316.7	50.3	32	401.7	99	42.2	133.1	18.6	16
*P-Value*	*0.000*	*0.000*	*0.000*	*0.000*	*0.000*	*0.000*	*0.000*	*0.02*	*0.08*
Total									
	44.5	3.9	12	41.4	4.8	15.6	32.9	6.4	12.2
N	4, 563			4,821			3, 473		

Similar to stunting levels, child wasting levels reduced with increasing maternal education in the three countries. Child wasting was statistically significantly associated with maternal education in Tanzania and Zimbabwe (p value = 0.02 and 0.07, respectively).

The prevalence of underweight in under-five children in Malawi, Tanzania and Zimbabwe was also negatively significantly associated with the mother’s educational attainment (p values <0.05).

### Logistic regression results

The likelihood ratio chi-square test and Pseudo R-square tests have been used to test the goodness of fit of the models. As Tables 3, 4 and 5 show the LR Chi-square test is significant at one percent across all the models, implying that the models fit well with the data. While the reference category for maternal education in the multivariate analyses was “no education” in Malawi and Tanzania, it was changed to ‘senior primary’ in Zimbabwe because there were few cases of women (only 4 percent) who had no education in Zimbabwe. Using ‘no education’ as the reference category would not be suitable for the case of Zimbabwe; The multivariate results, presented in Table 3, show that while maternal education is inversely related to child stunting, the results are only significant at high levels of education (secondary education and above) in all the three countries. The odds of stunting reduced in Malawi and Tanzania at the highest level of maternal education compared to no education. In Zimbabwe, the odds of child stunting reduced with increasing levels of maternal education compared to senior primary level, with significant results being obtained at the highest category of maternal education only. Other variables with significant relationship with child stunting were household wealth and child age (Table 3). The interaction between maternal education and other variables such as wealth was tried in our model but did not yield any significant result hence dropped in the final model.

**Table 3:** Relationship between stunting among children (0–59 months) with household, child, maternal characteristics in Malawi (2010), Tanzania (2009–10) and Zimbabwe (2005–6)

Dependent variable	Height- for-Age (% below -2SD) =1; 0 otherwise					
	Malawi		Tanzania		Zimbabwe	
	Odds ratio	t	Odds ratio	t	Odds ratio	T
Explanatory Variables						
Household Socioeconomic Factors						
Household head is female	0.94	−0.44	1.21	1.59	0.90	−1.05
Total number of people in the household	0.97	−1.43	0.99	−0.39	0.98	−0.66
Rural residence dummy	0.89	−0.81	1.05	0.39	1.12	0.56
Household Wealth	0.87	−3.45^b^	0.90	−2.39^c^	0.92	−1.10
Child Demographic Factors						
Age of child (in months)	1.02	7.57^b^	1.02	6.38^b^	1.01	3.07^b^
Whether the child is male	1.36	4.04^b^	1.50	5.56^b^	1.39	4.12^b^
Large size of the child at birth	0.48	−5.94^b^	0.56	−3.74^b^	0.66	−3.60^b^
Child is of multiple birth	2.75	2.56^b^	3.11	2.73^b^	2.67	2.79^b^
Birth order	1.05	1.46	1.07	2.30^c^	1.05	1.23
Maternal Demographic Factors						
Mother’s current age	0.98	−1.39	0.97	−2.31^c^	1.00	−0.36
Mother’s body mass - underweight	1.20	1.18	1.29	1.89	0.98	−0.14
Mother’s body mass - normal	1..00		1.00		1.00	
Mother’s body mass - overweight	0.69	−2.76^c^	0.83	−1.56	0.81	−1.90
Mother’s body mass – pregnant and postpartum	0.90	−0.86	0.79	−2.13^c^	0.73	−2.04^c^
Number of under-five children	1.05	0.77	1.02	0.42	1.04	0.49
Mother’s education level^a^						
Mother’s education – None	*1.00*		*1.00*		0.87	−0.59
Mother’s education – Junior Primary	0.85	−1.25	1.06	0.45	0.84	−0.78
Mother’s education – Senior Primary	0.82	−1.57	0.90	−1.02	*1.00*	
Mother’s education – Junior Secondary	0.89	−0.56	0.70	−1.50	1.03	0.28
Mother’s education – Senior secondary +	0.56	−2.24^c^	0.31	−4.00^b^	0.51	−2.00^c^
Household with safe water	0.91	−0.93	1.05	0.46	1.22	1.65
Household with improved toilet facility	1.03	0.26	0.74	−2.10^c^	1.10	0.52
F Statistic	6.70^b^		5.61^b^		3.16^b^	
LR χ^2^	294.68^b^		302.27^b^		122.22^b^	
Pseudo R^2^	0.04		0.05		0.03	
N	4,563		4,821		3,472	

Note: ^a^While the reference category for maternal education in the multivariate analyses was “no education” in Malawi and Tanzania, it was changed to ‘senior primary’ in Zimbabwe because there were few cases of women (only 4 percent) who had no education in Zimbabwe. Using ‘no education’ as the reference category would not therefore yield any significant results for Zimbabwe; ^b^significant at 1 % level; ^c^significant at 5 % level

**Table 4:** Relationship between wasting among children (0–59 months) with household, child, maternal characteristics in Malawi (2010), Tanzania (2009–10) and Zimbabwe (2005–6)

Dependent variable	Weight-for-Height (% below -2SD) =1; 0 otherwise					
	Malawi		Tanzania		Zimbabwe	
	Odds ratio	t	Odds ratio	t	Odds ratio	T
Socioeconomic Factors						
Household head is female	1.37	0.90	0.92	−0.27	0.84	−0.98
Total number of people in the household	1.08	1.36	1.01	0.40	0.84	−3.63^b^
Rural residence dummy	1.37	0.79	1.32	1.04	1.05	0.13
Wealth	0.97	−0.40	1.05	0.57	1.04	0.37
Child Demographic Factors						
Age of child (in months)	0.97	−4.45^b^	0.97	−4.16^b^	0.98	−3.96^b^
Whether the child is male	1.18	0.94	1.39	1.94	1.17	0.92
Large size of the child at birth	0.63	−1.75	0.61	−1.82	0.55	−3.27^b^
Child is of multiple birth	2.22	1.51	0.68	−0.71	1.47	0.88
Birth order	1.01	0.11	1.02	0.21	0.99	−0.10
Child Health Factors						
Had diarrhoea recently	0.84	−0.73	1.20	0.71	1.51	2.33^c^
Maternal Demographic Factors						
Mother’s current age	0.95	−1.57	1.00	0.03	1.02	1.06
Mother’s body mass - underweight	1.55	1.16	2.32	3.48^b^	1.53	1.80
Mother’s body mass - normal	1.00		1.00		1.00	
Mother’s body mass - overweight	0.65	−1.04	0.55	−1.94	1.05	0.21
Mother’s body mass – pregnant and postpartum	0.94	−0.20	1.11	0.46	1.31	1.21
Number of under-five children	0.76	−1.94	1.01	0.06	1.07	0.64
Mother’s education level^a^						
Mother’s education – None	*1.00*		*1.00*		0.79	−0.56
Mother’s education – Junior Primary	0.75	−1.19	0.95	−0.15	0.97	−0.09
Mother’s education – Senior Primary	0.69	−1.36	0.55	−2.76^b^	*1.00*	
Mother’s education – Junior Secondary	0.43	−1.75	0.33	−2.24^c^	0.61	−2.22^c^
Mother’s education – Senior secondary +	0.34	−2.04^c^	0.33	−1.98^c^	0.15	−240^c^
Household with safe water	0.82	−0.74	1.03	0.14	0.96	−0.22
Household with improved toilet facility	1.25	0.77	1.04	0.19	0.85	−0.74
F Statistic	3.40^b^		4.74^b^		3.52^b^	
LR χ^2^	127.65^b^		138.49^b^		76.35^b^	
Pseudo R^2^	0.06		0.07		0.05	
N	4,531		4,739		3,473	

Note: ^a^While the reference category for maternal education in the multivariate analyses was “no education” in Malawi and Tanzania, it was changed to ‘senior primary’ in Zimbabwe because there were few cases of women (only 4 percent) who had no education in Zimbabwe. Using ‘no education’ as the reference category would not therefore yield any significant results for Zimbabwe; ^b^significant at 1 % level; ^c^significant at 5 % level

**Table 5:** Relationship between underweight among children (0–59 months) with household, child, maternal characteristics in Malawi (2010), Tanzania (2009–10) and Zimbabwe (2005–6)

Dependent variable	Weight-for-Age (below -2SD)					
	Malawi		Tanzania		Zimbabwe	
	Odds ratio	T	Odds ratio	T	Odds ratio	T
Explanatory Variables						
Child Demographic Factors						
Age of child (in months)	1.01	2.26^c^	1.01	1.69	1.00	1.67
Whether the child is male	1.29	2.20^c^	1.50	4.01^b^	1.24	1.31
Large size of the child at birth	0.38	−6.70^b^	0.40	−5.54^b^	0.47	−4.87^b^
Child is of multiple birth	2.27	2.35^c^	2.65	3.98^b^	2.09	2.51^c^
Birth order	1.05	0.94	1.05	1.07	1.03	0.62
Child Health Factors						
Had diarrhoea recently	1.58	3.28^b^	1.19	1.22	1.54	2.84^b^
Maternal Demographic Factors						
Mother’s current age	0.98	−1.13	0.99	−0.36	1.00	0.23
Mother’s body mass - underweight	1.77	2.87^b^	1.96	3.89^b^	1.89	3.29^b^
Mother’s body mass - normal	1.00		1.00		1.00	
Mother’s body mass - overweight	0.57	−2.32^b^	0.54	−3.23^b^	0.76	−1.51
Mother’s body mass – pregnant and postpartum	0.98	−0.14	0.97	−0.21	0.76	−1.25
Number of under-five children	1.09	0.93	1.06	0.77	1.28	2.59^c^
Socioeconomic Factors						
Household head is female	1.62	2.18^c^	1.42	1.75	0.90	−0.79
Total number of people in the household	0.97	−0.84	0.98	−0.81	0.92	−2.73^b^
Rural residence dummy	0.78	−1.17	0.95	−0.28	1.00	−0.01
Wealth	0.38	−0.96	0.87	−2.09^c^	0.95	−0.62
Mother’s education level^a^						
Mother’s education – None	*1.00*		*1.00*		0.95	−0.14
Mother’s education – Junior Primary	0.78	−1.49	0.91	−0.43	0.68	−1.30
Mother’s education – Senior Primary	0.77	−1.46	0.79	−1.71	*1.00*	
Mother’s education – Junior Secondary	0.46	−2.34^c^	0.57	−1.58	0.85	−1.22
Mother’s education – Senior secondary +	0.45	−1.99^c^	0.40	−2.18^c^	0.12	−2.78^b^
Household with safe water	0.92	−0.57	0.84	−1.46	1.41	1.73
Household with improved toilet facility	0.84	−0.97	0.88	−0.83	0.86	−0.70
F Statistic	4.79^b^		3.65^b^		4.70^b^	
LR χ^2^	154.63^b^		170.45^b^		139.16^b^	
Pseudo R^2^	0.05		0.04		0.06	
N	4,563		4,821		3,473	

Note: ^a^While the reference category for maternal education in the multivariate analyses was “no education” in Malawi and Tanzania, it was changed to ‘senior primary’ in Zimbabwe because there were few cases of women (only 4 percent) who had no education in Zimbabwe. Using ‘no education’ as the reference category would not therefore yield any significant results for Zimbabwe; ^b^significant at 1 % level; ^c^significant at 5 % level

Similar to the results on chid stunting, maternal education was inversely related to wasting but the results are significant only at high levels of education (Table 4). In particular, while higher levels of maternal education are associated with reduced wasting compared to no education, the results are only significant at senior secondary and above level in Malawi, at senior primary level and above in Tanzania and at junior secondary level and above in Zimbabwe. The interaction between maternal education and wealth did not yield statistically significant result and was finally dropped in the final model.

Consistent with results from the previous two models, the odds of child underweight were reduced at higher levels of maternal education compared to no education in Malawi and Tanzania, but the results were significant at junior secondary level and above in Malawi and senior secondary level and above in Tanzania (Table 5). In, the odds of being underweight among children in Zimbabwe reduced with increased levels of maternal education compared to senior primary level. It is important to note that, as was with stunting and wasting, there was no interaction between maternal education and wealth.

### Maternal education threshold level

The multivariate results for the three measures of child nutritional status shown that while the education of the mother is an important determinant of the nutritional status of their children, the relationship is statistically significant only at high levels of education. Table 6 summarizes the associations of the different levels of mother’s education on the three anthropometric indicators and depicts the estimated threshold levels of maternal education for the three measures of child nutritional status. For stunting, the influence of increasing maternal education is only being seen at the senior secondary and above in all the three countries. In the case of wasting, maternal education is showing a significant impact at the senior primary level and above in Tanzania, at junior secondary level and above in Zimbabwe and at the senior secondary and above in Malawi. These results imply that that lower levels of maternal education do not seem to have any significant associations with reduced odds of child wasting. Similarly, maternal education is significantly associated with reduced odds of being underweight at junior secondary level and above in Malawi and Tanzania and at the highest category of maternal education in Zimbabwe (Table 6). The multivariate results show that the threshold level of maternal education that is necessary to make significant reduction in child malnutrition is at least junior secondary school level (at least 9 years of schooling) in Malawi; senior primary school level (at least 5 years of schooling) in Tanzania and; junior secondary school level in Zimbabwe (at least 8 years of schooling) (Table 6). These minimum threshold levels are derived from the wasting and underweight models which showed significant relationship between maternal education and wasting at relatively low levels of maternal education. If we consider the stunting as most commonly used measure of child nutritional status, the minimum threshold level rises to senior secondary level and above (at least 11 years of schooling) in all the three countries. Below these threshold levels, maternal education has no significant positive influence on child stunting.

**Table 6:** Threshold levels of maternal education

Education Category	Malawi			Tanzania			Zimbabwe		
	Stunting	Wasting	Under-weight	Stunting	Wasting	Under-weight	Stunting	Wasting	Under-weight
No education	RC	RC	RC	RC	RC	RC			
Junior Primary									
Senior Primary					*		RC	RC	RC
Junior Secondary			*		*	*		*	
Senior Secondary+	*	*	*	*	*	*	*	*	*

Note: RC means reference category *means statistically significant

## Discussion

This paper investigates whether there is a threshold level of maternal education necessary to reduce child undernutrition. The study shows that maternal education is important in addressing child undernutrition in the three countries. Bivariate results show negative significant association between maternal education and the three measures of child nutritional status in all the three countries. Multivariate analysis also shows that higher levels of maternal education are required for accruing positive influence on child nutritional status. Using stunting as the most widely acceptable measure of child growth faltering, the minimum threshold level of maternal education that is necessary to reduce stunting is senior secondary and above (i.e. more than 10 years of schooling in all the three countries). Contrary to findings of Hobcraft [17] and Mensch [18], our findings point to the fact that there is a threshold level of maternal education below which the education of the mother does not have any significant influence on child nutrition.

These results are consistent with the literature on the association between maternal education and child health and nutrition [1], [19–22]. At relatively high levels of maternal education, mothers tend to have acquired the necessary health knowledge and are more able to practice recommended feeding practices for their children [11, 23]. Furthermore, relatively educated women tend to have relatively fewer children and are able to provide better care and support to their children, all of which positively impact on children’s nutritional outcomes. Cleland [15] argued that maternal education has a strong influence on early childhood health and survivor outcomes majorly through economic advantages associated with education. In countries covered by this study, access to health information, as well as health services are limited. Mothers who are educated beyond junior secondary school level are therefore more likely to have a higher maternal diagnostic ability of child growth performance and are therefore able to take corrective action to address any cases of child undernutrition.

In the context of southern and eastern Africa, women who have studied beyond primary education tend to have increased command over household resources, enabling them to make significant contributions towards the promotion of their children’s nutrition and health status. Such women are more able to participate in income generating activities that improve household incomes and their ability to provide better nutrition for their children. In the rural areas of countries like Malawi, Tanzania and Zimbabwe, informal group lendings and village savings and loan groups are common among women and most of these are patronized by relatively educated women. Through these groupings, women are able to raise income to promote household food security and nutrition.

This analysis contributes to a better understanding of the specific threshold of maternal education that has a positive outcome on childhood undernutrition using nationally representative data from three sub-Saharan African Countries. Based on the findings of this study, the following are implications for policy; In all the three countries, if maternal education is to play a significant role in reducing child malnutrition, women need to be educated beyond the primary school level. While there is free primary school education in all the three countries, having been introduced in 1994 in Malawi; 2002 in Tanzania; and 1980 in Zimbabwe, this alone may not be sufficient in creating a positive contribution towards improved child undernutrition. The threshold level of maternal education in all the three countries is beyond the primary school level. The level of maternal education is known to be an important predictor of child stunting even in informal settlements [24]. Besides the link between maternal education and socio-economic status pathways that influence child nutrition, [11] access to health care and access to money [25] are other predictors of child nutrition. Policies to ensure that girls remain in school beyond the primary school level, therefore, hold more promise in addressing child nutritional problems in the three countries. According to the Malawi Ministry of Education Science and Technology, [26], girls’ secondary school enrolments in 2011 were only 5.6% compared to 6.9% for boys, signifying the large number of female primary school leavers that do not make it into secondary school in Malawi. Therefore, policies to improve the enrolment of girls at secondary school level, especially in Malawi and Tanzania would improve maternal education of future mothers and contribute towards promoting child nutrition in future. The use of cross-sectional data which is limited in demonstrating the direction of relationships of variables. However, the national coverage of data gives the power for generalizability of findings. The study does not investigate women autonomy in control for money and household decision making which may have implications for child health and nutrition outcomes.

## Conclusion

In all the three countries, maternal education is having a significant influence on child stunting, when the mother has at least a senior secondary level of education. Since low height-for-age is associated with poor socioeconomic conditions, frequent illnesses and poor feeding practices, women’s educational levels beyond junior primary school in the three countries has the potential to reduce the suboptimal health and nutritional conditions of their children. Increased investment in women’s education beyond primary school level is a promising intervention in countries like Malawi and Tanzania, which have a high burden of child undernutrition. We recommend a further analysis of data using a stepped regression model to demonstrate how inclusion of other important variables would affect the impact of maternal education to child nutrition at various levels of in each country.

## Ethical approval

The DHS protocol is approved by the ICF Macro ethical review board and specific county review boards. Approval for data use was provided by ICF macro. Informed consent was obtained from each of the respondents interviewed in DHS surveys.
